# Sex-based differences in biomechanical function for chronic low back pain and how it relates to pain experience

**DOI:** 10.1007/s00586-025-08730-2

**Published:** 2025-03-20

**Authors:** Erin Archibeck, Irina Strigo, Aaron Scheffler, Abel Torres-Espin, Karim Khattab, Pavlos Silvestros, Robert Matthew, Caitlin Regan, Paul Hodges, Conor O’Neill, Jeffrey Lotz, REACH Inverstigators, Jamie Ahn, Kristina Benirschke, Alexandra Bryson, Katherine Bunda, Briana Davis, Carolina Dorofeyev, Rosalee Espiritu, Pirooz Fereydouni, Aamna Haq, Nicholas Harris, Sara Honardoost, Gabriel Johnson, Jennifer Johnson, Edward Lingayo, Robert Miller, Phirum Nguyen, Christopher Orozco, Lindsay Ruiz-Graham, Kie Shidara, Kaitlyn Smith, John Boyuan Xiao, Michelle Yang, Grace O’Connell, Jeannie Bailey

**Affiliations:** 1 University of California, Berkeley, Berkeley, USA; 2 University of California, San Francisco, San Francisco, USA; 3 University of Waterloo, Waterloo, Canada; 4 University of Queensland, Brisbane, Australia

**Keywords:** Biomechanics, Motion capture, Motion analysis, Pain experience, Quantitative sensory testing, Kinematics, Composite score, Chronic low back pain

## Abstract

**Purpose:**

The relationship between pain experience and biomechanical impairment in chronic low back pain (LBP) is unclear. Among the broader pain literature, sex-based differences in pain experience have been established. However, it is unknown if sex-based differences in pain experience relates to compromised movement patterns for patients with chronic LBP. This study examined sex differences and whether there are sex-based associations between pain experience and biomechanical function in patients with chronic LBP.

**Methods:**

To capture the biomechanical variability among LBP patients, we quantified full-body movement quality based on the extent that 3D postural trajectories deviated from matched controls during a sit-to-stand task (Kinematic Composite Score, K-Score). For both males and females, the K-Score was compared to pain measures, including patient-reported metrics and quantitative sensory testing (pressure pain threshold, PPT).

**Results:**

There were significant sex-based differences in pain experience and biomechanical function in patients with LBP. Specifically, males exhibited ~ 8% lower trunk K-Scores, indicating biomechanical function that deviated more from controls when compared to female participants (*p* < 0.001). However, females exhibited PPT values 29% and 41% lower than males at the control and pain sites, respectively (*p* < 0.0001). There was a weak but significant negative association between PPT and K-Scores for males (R^2^ = 0.14, *p* < 0.01), while females lacked an association.

**Conclusion:**

Overall, males with LBP exhibited worse movement quality, driven by trunk motion, but higher PPTs. Possible explanations include reduced interoceptive awareness or increased kinesiophobia in males, which may influence movement patterns. This research is an initial step in uncovering the complex relationship between patient-specific factors influencing LBP disability, laying the groundwork for further exploration, and paving the way for improving outcomes with patient-specific treatments.

## Introduction

Chronic low back pain (LBP) is the leading cause of global disability and the number one healthcare expenditure in the United States [1]. Approximately 90% of chronic LBP is nonspecific, lacking clear anatomical pathology, and exhibiting tremendous heterogeneity in movement impairment [2]. Further, treatment outcomes have seen minimal improvements over the past 3 decades [3], underlining the critical need to study patient-specific factors, including biological sex, that may inform the variability in experienced pain and biomechanical impairment among chronic LBP patients.

Over the past two decades, there has been a growing interest in sex-based differences in pain perception [4]. Pressure pain threshold (PPT), which represents the minimum pressure required to induce pain, as well as patient-reported outcomes can be used to estimate pain experience. Notably, there are no observed sex-based differences in PPT in the lumbar region of healthy subjects [5]. However, for chronic LBP patients, PPT values were 32% lower in female patients when compared to male patients, indicating greater pain sensitivity [6]. Additionally, patient-reported outcomes underline sexual dimorphism in musculoskeletal pain experience, with female patients generally reporting greater pain severity, pain interference, and pain anxiety [7–11]. Mechanisms for this difference include biological, psychological, and sociocultural factors [8, 12, 13].

While there may be fundamental differences in pain experience between males and females, there is a gap in knowledge regarding whether these sex-based differences translate to differences in biomechanical behavior. A plethora of research has highlighted the importance of continued exploration into the interface of pain and movement [14–17], but sex-based differences in this relationship remain less understood. Although sensitivity to mechanical stimuli in females is higher, studies report that males with chronic pain have lower activity levels [18, 19] and greater kinesiophobia [20–23] compared to females. Additionally, prior studies showed sex-based differences in biomechanical features among individuals with LBP, encompassing differences in pelvic and leg motion such as lumbopelvic angular displacement and pelvic, knee, and ankle range of motion [24–27]. However, the clinical relevance of these differences remains unclear, possibly due to the use of isolated biomechanical parameters (e.g., peak joint velocity), which overlook the comprehensive aspect of dynamics and function in heterogeneous biomechanical impairments. For this reason, we employed the Kinematic Composite Score (K-Score), an alternative approach that captures overall movement variability, offering a more comprehensive assessment of biomechanical function in patients with LBP.

It is uncertain whether sex differences in pain experience translate to differences in biomechanical function. To address the variability and limited characterization of biomechanical impairments in LBP, the K-Score was used to quantify deviations in full-body movement compared to healthy controls. We compared the biomechanical function with patient-reported metrics and quantitative sensory testing. We hypothesize that males with chronic LBP will have lower levels of pain sensitivity, pain interference, and pain-related anxiety compared to females. Furthermore, we expect this reduced pain experience in males with LBP to be associated with superior biomechanical function.

## Methods

Patients with chronic low-back pain (LBP, n = 194) were selected from the IRB-approved comeBACK study (IRB #20,204,648), recruited from four University of California medical centers (San Francisco, Davis, San Diego and, Irvine). The study followed the Declaration of Helsinki, and all participants gave informed consent. Patients with LBP for over 3 months and more than 50% of days were included. Additionally, age-matched controls (CTRL, n = 62) with no LBP in the past 24 months were selected via community recruitment and used as a reference for K-Score calculations and PPT, with our primary focus on sex-based differences within the LBP group. Both groups excluded individuals with significant comorbidities, spine or lower extremity surgeries, referral pain, BMI > 35, or inability to walk unaided.

### Pain outcomes

Patient-reported metrics were collected to assess perceived pain in LBP patients. Pain anxiety was measured using the modified Pain Anxiety Symptom Scale (PASS-20) [28], ranging from 0 to 100, with 100 representing extreme anxiety. Pain interference was assessed with the PROMIS Pain Interference Score (PROMIS-PI) [29], ranging from 4 to 20, with higher scores indicating more interference. Mechanical quantitative sensory testing, conducted in the hospital testing room and administered by trained study coordinators, assessed pain sensitivity for both CTRL and LBP groups [30]. A pressure algometer (Wagner FPK20 with 1 cm^2^ rubber tip) applied increasing pressure at a rate of 49 kPa/s at a pain and control site (1500 kPa max, metronome guided). Participants were instructed to indicate the onset of pain by stating "stop" immediately upon perceiving any discomfort. Then, the algometer output was recorded as the pressure pain threshold (PPT, kPa). The pain site, located near the lumbar erector spinae muscle, was identified by the participant’s response to manual over-pressure (springing palpation) performed in the prone position. The control site was in the upper trapezius contralateral to pain site. Every participant was familiarized with the procedures prior to testing. Three repetitions were completed with 30 s rest intervals between each repetition and pain threshold values were averaged for each patient. The probe placement was varied slightly trial to trial to prevent sensitization.

### Biomechanical assessment and kinematic analysis for movement quality

**Fig. 1:**
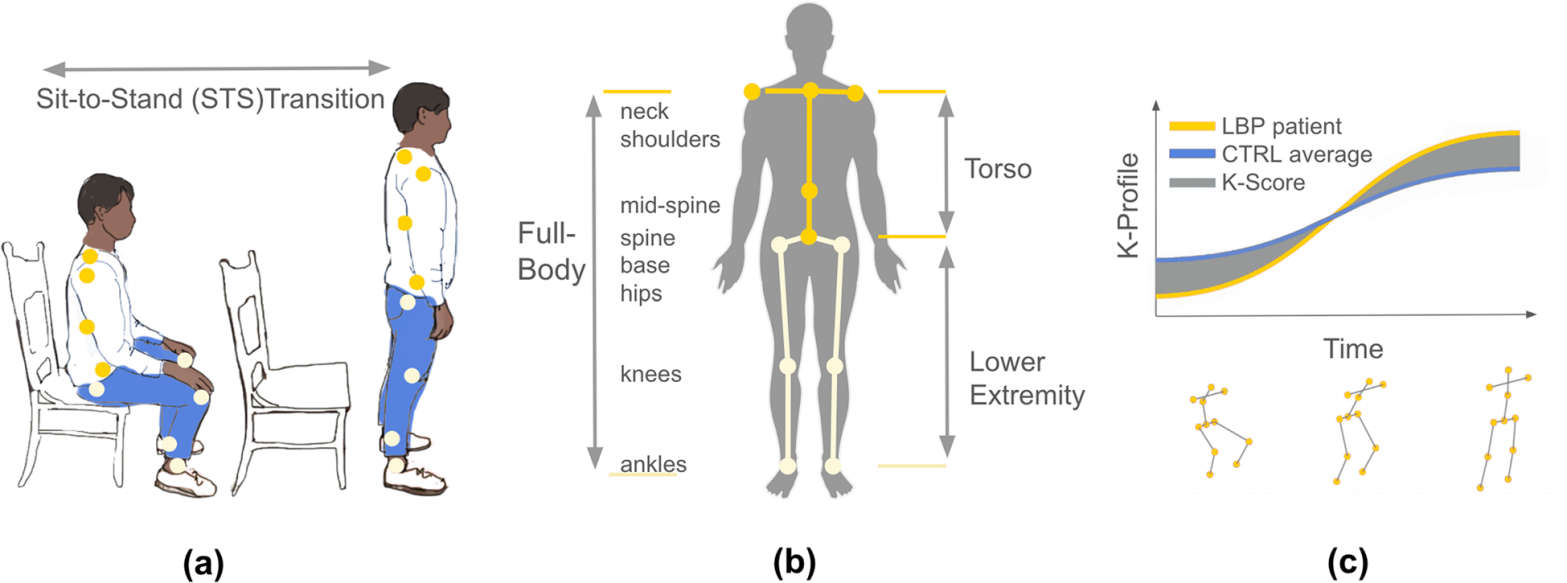
Schematic of the motion capture and metric methodology: **a** STS transition period, **b** Kinect torso (dark yellow) and lower extremity (light yellow) landmarks, and **c** Kinematic Profile (K-Profile) and Kinematic Composite Score (K-Score)

Full-body markerless motion capture (Azure Kinect, Microsoft) estimated position data (sampling frequency rate = 30 Hz), including 5 landmarks on the trunk (neck, left and right shoulders, mid-spine, base of the spine) and 6 on the lower extremities (left and right hips, knees, and ankles) (Fig. 1b) during five sit-to-stand (STS) maneuvers (Fig. 1a). STS is a relevant functional task characterized by full-body involvement of the trunk and lower extremities [30, 31]. Participants performed the task using a standard 17-inch height chair with arms by their side and feet placed hip-width apart. Participants were instructed to move at a natural and comfortable pace, with timed audio cues provided between each motion.

The Kinematic Profile (K-Profile), and Kinematic Composite Score (K-Score), a novel approach for analyzing full-body biomechanics, were used to assess movement during five STS transition repetitions, defined as the motion from a seated to standing position, excluding any pauses (Fig. 1a). The first trial was removed from analyses, as preliminary results showed greater variability compared to the following four repetitions. Data for the subsequent four trials was temporally normalized using min–max scaling. Principal Component Analysis (PCA) was performed using Python scikit-learn library by encompassing landmark positions for every time point. Generalized Procrustes Analysis was applied to the PCA-transformed data for standardization, with the control average chosen as the reference frame to ensure that the scores remained consistent despite including patient-specific data. The use of PCA and Generalized Procrustes Analysis for quantifying 3D motion capture data was inspired by previous work in our lab [32].

The K-Profile, capturing the most prominent patterns across all landmarks, was calculated using the weighted sum of the PC scores at every time point. The Kinematic Score (K-Score), was established by calculating the total difference between the individual’s score and the CTRL average, an approach commonly used in developing kinematic scores [33, 34]. To enhance comprehensibility, all values were transformed such that 100 represents the CTRL average movement trajectory. The K-Score allows insight into the extent of alignment to the “ideal trajectory”, defined by the CTRL group with no pain or reported biomechanical impairment. Hence, in this study, superior “movement quality” is defined as movement that aligns with the healthy CTRL group average. Given that the STS transition phase represents the primary motion engaging the body across the trunk and lower extremities, trunk (tK-Score) and lower extremity (leK-Score) K-Scores were also calculated to study respective contributions of the body segments (Fig. 1b). Full-body, trunk, and lower extremity K-Scores were compared between patient groups at all repetitions.

### Outcomes and statistics

Linear mixed-effects models were constructed to assess the relationship between the K-Scores and predictors (repetition and sex) using the R package nlme, for both CTRL and LBP groups. Variance of K-scores differed across all groups to account for heteroskedasticity. Further, a random intercept term for each patient accounted for potential correlations within individual patients. An F-test confirmed that the inclusion of the interaction between repetitions and patient type improved the model. Linear contrasts compared groups at every repetition using the R package *multcomp*, with adjustments made for multiple comparisons. K-Scores are reported as mean ± standard deviation(SD). Significance was assumed for *p*-values < 0.05. Given that the data did not follow a normal distribution as confirmed with Shapiro–Wilk, nonparametric analysis of covariance (ANCOVA-R package car) [35] was used for patient-reported metrics and PPT, correcting for age and BMI, with results reported as median ± interquartile range (IQR).

Multiple regression analysis explored the correlations between biomechanical function (K-scores) as the outcome and predictors including age, BMI, and pain (PASS-20, PROMIS-PI, PPT), with Spearman rank transformations applied to the predictor variables. Each pain measure was analyzed separately using its own regression model. The coefficient of determination (R^2^) was used to determine whether correlations were weak (R^2^ < 0.16), moderate (0.16 < R^2^ < 0.36) or strong (R^2^ > 0.36) [36, 37].

## Results

### Demographics and pain outcomes

**Fig. 2:**
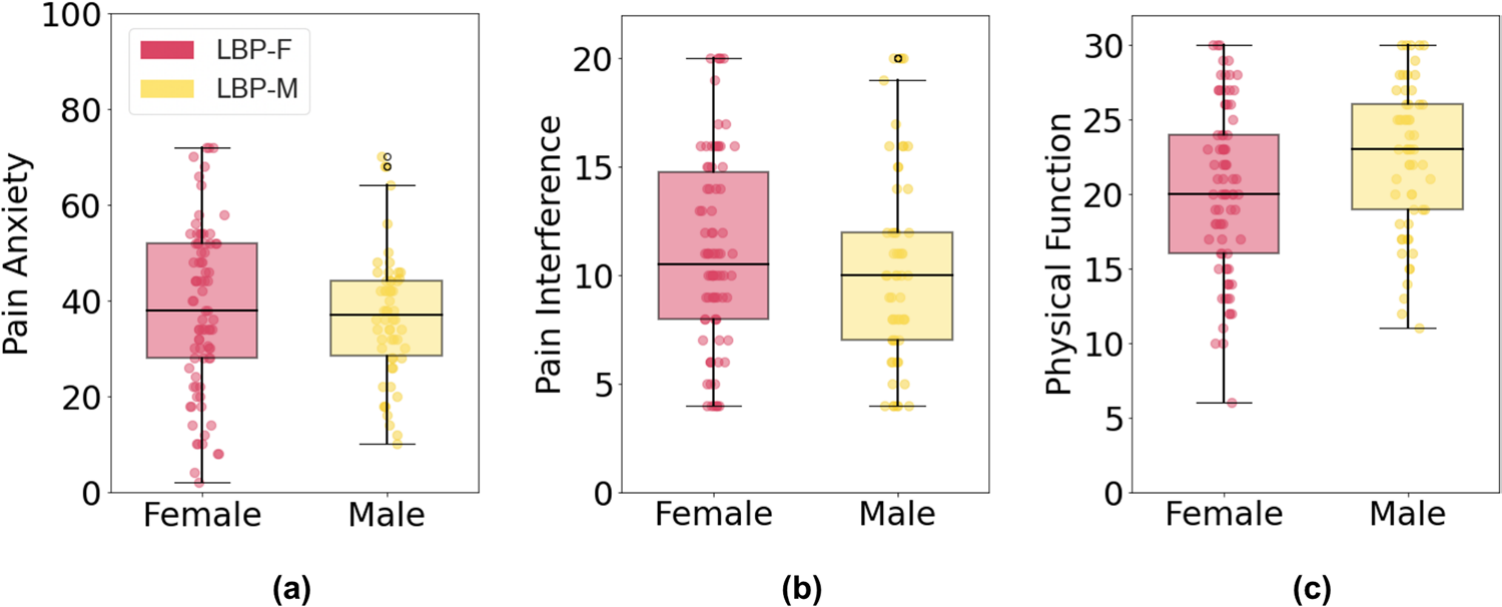
Patient-reported pain outcomes for **a** pain anxiety scores (PASS-20), and **b** pain interference (PROMIS-PI) for patients with LBP. No statistical differences were observed (*p* > 0.25)

LBP-F females (LBP-F; n = 110; 55 ± 15 years) and LBP-M males (LBP-M; n = 84; 56 ± 16 years) were comparable in age and BMI (*p* = 0.77 and 0.92, respectively). There was no sex-based difference in pain anxiety (PASS-20; *p* = 0.5; Fig. 2a) or pain interference (PROMIS-PI; *p* = 0.37; Fig. 2b) (Table 1).

**Table 1:** Patient reported pain outcomes (PASS-20, PROMIS-PI), pressure pain threshold (PPT), and K-Scores (full-body, trunk, lower extremity) for females and males with LBP

Measurement		Female	Male	*p*-value
Patient-Reported Pain Outcomes (median ± IQR)	PASS-20 (0–100)	38.0 ± 24.0	37.0 ± 15.5	0.5
	PROMIS-PI (4–20)	10.5 ± 6.8	10.0 ± 5.0	0.37
Pressure Pain Threshold (kPa) (median ± IQR)	Control Site	295 ± 120	418 ± 298	< 0.0001
	Pain Site	291 ± 236	497 ± 434	< 0.0001
Full-body K-Score (mean ± SD)	Repetition 2	84.1 ± 7.8	79.1 ± 5.9	< 0.0001
	Repetition 3	83.8 ± 9.2	79.6 ± 9.7	0.002
	Repetition 4	83.6 ± 9.0	80.2 ± 7.7	0.004
	Repetition 5	84.4 ± 7.5	81.3 ± 6.2	0.002
Trunk K-Score (tK-Score) (mean ± SD)	Repetition 2	79.3 ± 5.9	74.8 ± 4.5	< 0.001
	Repetition 3	79.5 ± 5.3	74.5 ± 5.0	< 0.001
	Repetition 4	78.9 ± 5.5	73.8 ± 4.8	< 0.001
	Repetition 5	78.9 ± 5.6	73.6 ± 4.5	< 0.001
Lower Extremity K-Score (leK-Score) (mean ± SD)	Repetition 2	82.9 ± 4.8	81.4 ± 6.9	0.06
	Repetition 3	81.7 ± 4.9	80.4 ± 5.7	0.07
	Repetition 4	81.5 ± 7.8	82.1 ± 6.0	0.52
	Repetition 5	82.5 ± 4.2	82.1 ± 3.7	0.49

**Fig. 3:**
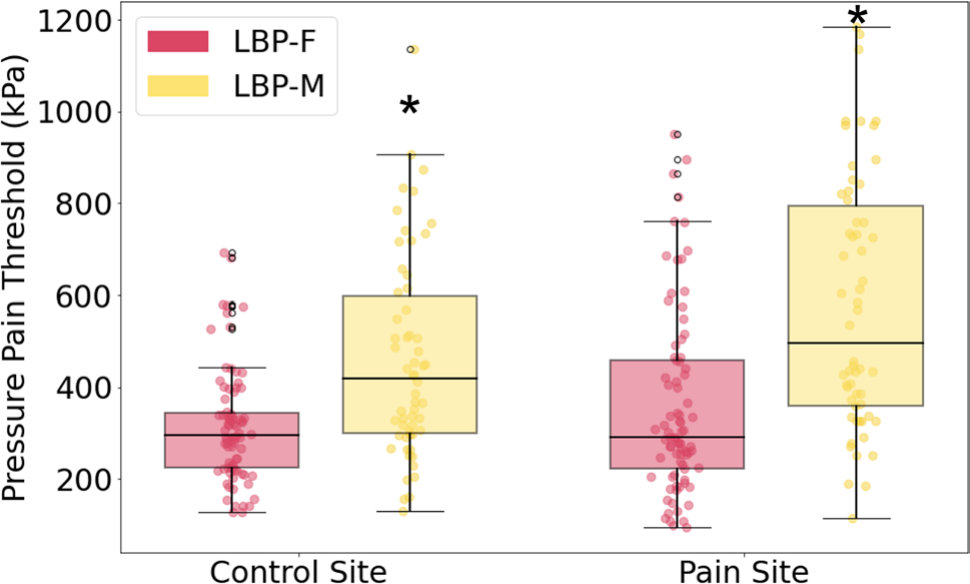
Mechanical quantitative sensory testing for patients with LBP. *represents *p* < 0.0001 between LBP-F and LBP-M

QST results revealed no sex-based difference in PPT for the healthy CTRL cohort in the shoulder (CTRL-F: 473 ± 283, CTRL-M: 580 ± 455 kPa; *p* = 0.07) or low back locations (CTRL-F: 550 ± 396, CTRL-M: 700 ± 444 kPa; *p* = 0.08), but overall PPT was higher in CTRLs compared to LBP groups. However, sex-based differences in PPT for the LBP group were observed at both the control and pain site. PPT was 29% lower for female participants than male participants at the control site (*p* < 0.0001; Table 1) and 41% lower at the pain site (*p* < 0.0001; Table 1) (Fig. 3).

### Biomechanics

**Fig. 4:**
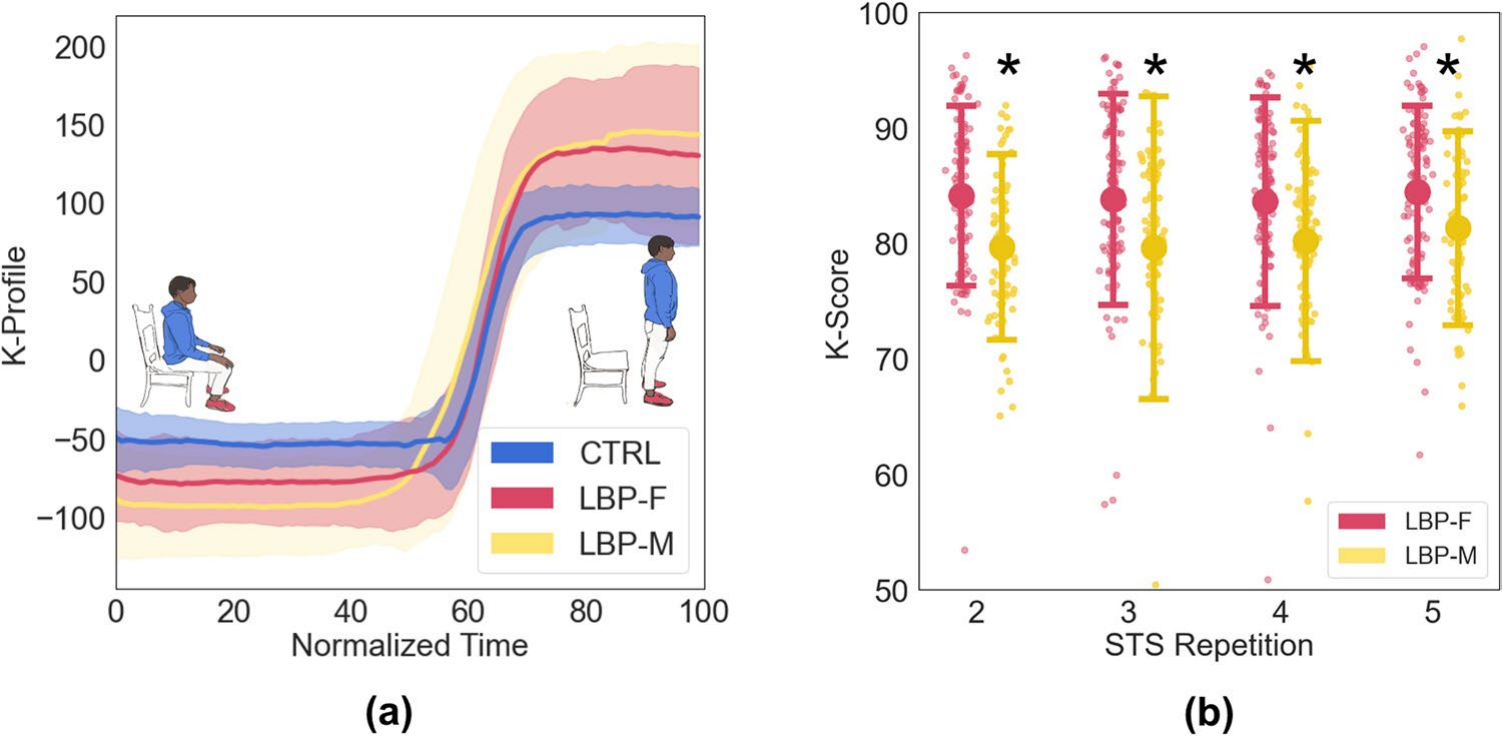
**a** K-Profile for repetition 2, and **b** K-scores for repetitions 2–5. *represents *p* < 0.005 between LBP-M and LBP-F

Participants in the CTRL group (n = 62, age = 58 ± 21; 32% male) exhibited minimal variation in movement patterns (K-Profile; blue shaded in Fig. 4a) and movement quality (K-Score; Fig. 4b) between participants and repetitions. Due to no sex-based differences in CTRL K-Scores (*p* > 0.3, (K-Score full body mean difference = 0.02, 95% CI: −2.32 to 2.34) and in effort to reduce variability and improve direct sex-based comparisons of the LBP group, data for all CTRLs were pooled and used as the “ideal trajectory” for the K-Score algorithm. For all repetitions, both LBP groups exhibited significantly lower K-Scores compared to CTRLs (p < 0.001). LBP-M exhibited ~ 5% lower full-body K-Scores compared to LBP-F across the four repetitions (*p* < 0.0001; Fig. 4b; Table 1), highlighting motion in males that deviated further from the healthy control average compared to females.

**Fig. 5:**
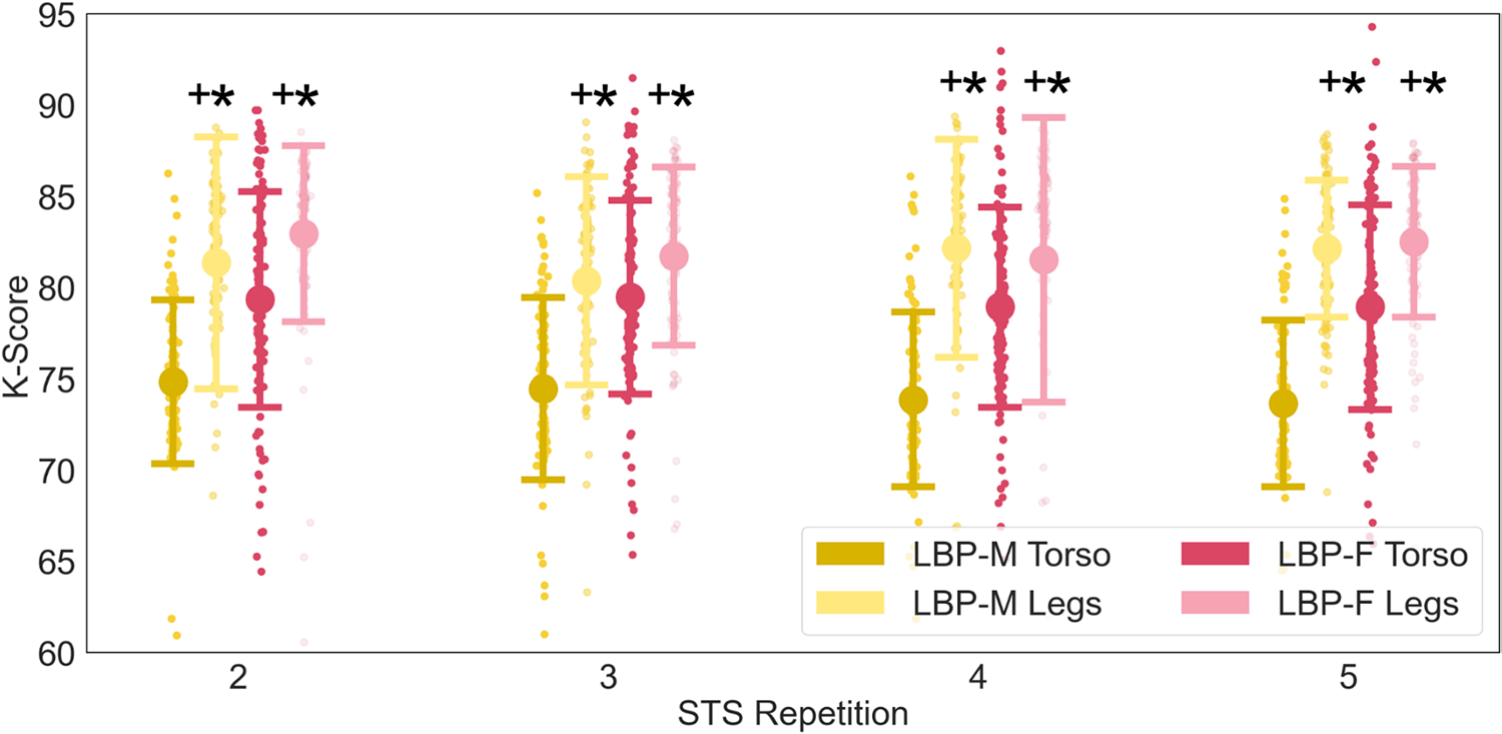
Torso K-Scores (tK-Scores) and lower extremity K-Scores (leK-Scores). + represents *p* < 0.01 between leK-Scores and tK-Scores within the same group, and *represents *p* < 0.001 between LBP-F and LBP-M

With respect to body segment (trunk and lower extremity) analysis, for within-subject comparisons, tK-Scores were ~ 5% lower than leK-Scores for all four repetitions (*p* < 0.001). tK-Scores for the LBP-M group was ~ 8% lower than LBP-F tK-Scores (*p* < 0.001; Table 1).There was no significant difference between LBP-M and LBP-F for leK-Scores for any STS repetition (*p* > 0.1) (Fig. 5).

### Relationship between pain measures and biomechanics

**Fig. 6:**
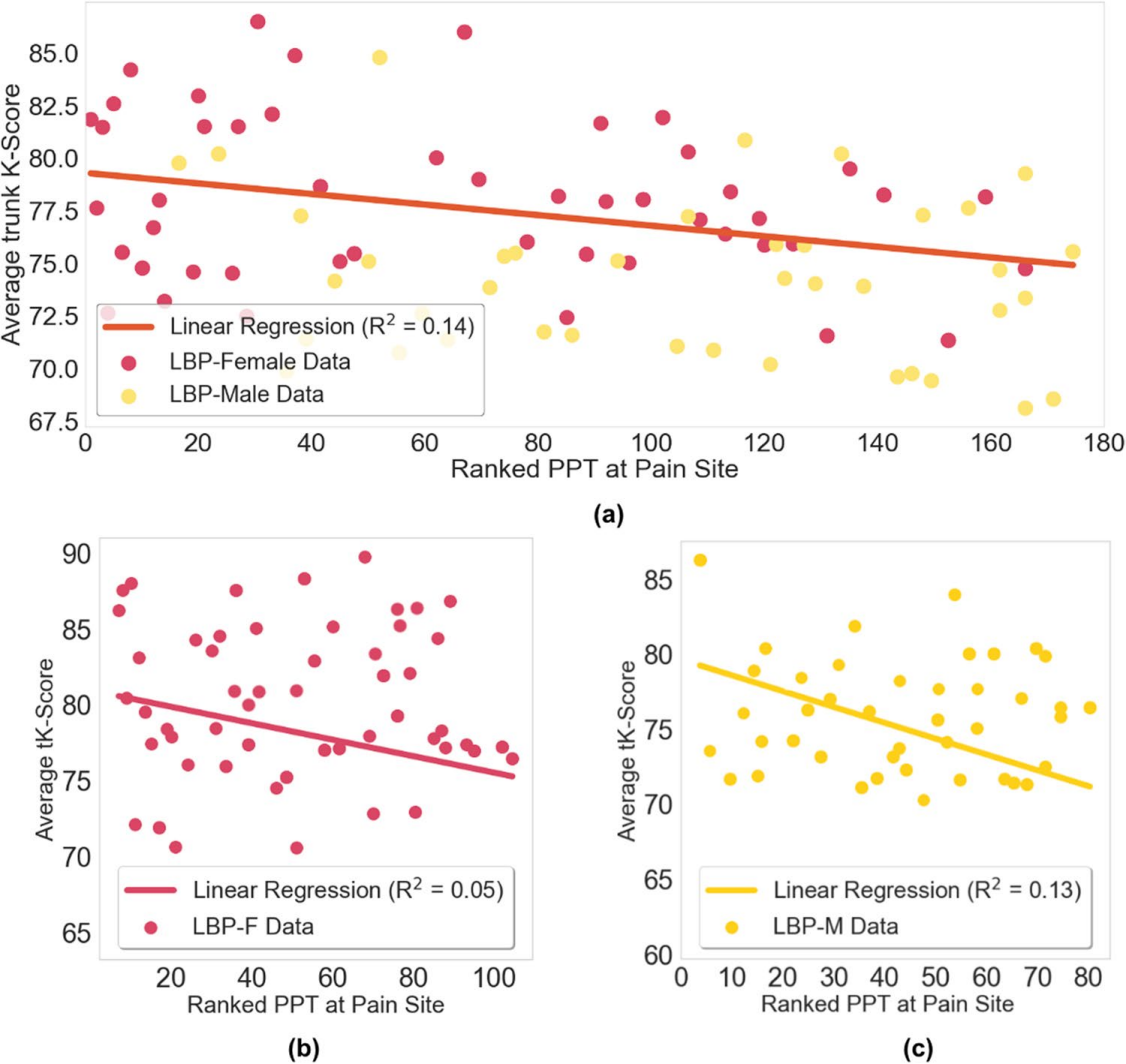
Multiple regression models for averaged tK-Scores and ranked PPT: **a** all LBP data (R^2^ = 0.14, *p* < 0.01), **b** LBP-F (R^2^ = 0.05, *p* = 0.29), and **c** LBP-M (R^2^ = 0.13, *p* = 0.02)

In the regression models using tK-Scores as the outcome, no significant correlations were found with the patient-reported metrics PASS-20 (R^2^ = 0.001, *p* = 0.6) or PROMIS-20 (R^2^ = 0.008, *p* = 0.2) as predictors. However, there was a weak but significant association between tK-Scores and PPT after adjusting for age and BMI (R^2^ = 0.14, *p* < 0.01; Fig. 6a). When split by sex, no significant correlation was found for the LBP-F group (R^2^ = 0.05, *p* = 0.29; Fig. 6b), but the LBP-M group showed a weak negative correlation (R^2^ = 0.13, *p* = 0.02; Fig. 6c).

## Discussion

This study investigated biomechanical function during repeated STS motion and pain experience in chronic LBP patients. Males with LBP exhibited lower pain sensitivity compared to females at both pain and control sites. While we hypothesized lower pain sensitivity in males, this was unexpectedly associated with worse biomechanical function. Males with LBP had lower movement quality and higher variability, largely due to trunk motion (tK-Score). No sex differences in K-Score were found in controls, suggesting LBP-induced sex differences in biomechanical function. These results highlight sex-based distinctions, informing the mechanistic relationship between pain and biomechanical impairment among chronic LBP patients.

Despite distinct sex-based differences in movement quality, patient-reported metrics (PASS-20, PROMIS-PI) were unable to discern sex-based differences. Rather, assessment of PPT, a tool to measure a patient’s perception of local pain responses, offers insight into sex-based distinctions of pain experience [10]. Our results, as well as previous research, has confirmed that patients with chronic LBP often exhibit lower PPT compared to healthy controls [5, 10]. Both our findings and additional studies suggest that there are no significant sex-based differences in PPT in the lumbar region among healthy individuals [5, 6]. However, PPT values in the lumbar spine decrease by 15–47% in LBP patients compared to controls, with females showing larger decreases [6]. Additionally, females with LBP had lower PPT at the tibialis anterior muscle [6]. Data from this study supported the notion that females with LBP exhibit lower PPT in both their lumbar region (41% lower compared to males) and secondary locations (shoulder; 29% lower compared to males). These findings suggest that these differences are not limited to structural issues in the spine and more indicative of general full body heightened pain sensitivity in females.

PPT provided a negative association with biomechanical quality, explaining 14% of the variability in tK-Scores across all participants. While the associations in this study were weak, they highlight complexity of pain and movement, emphasizing the value of identifying nuanced associations between pain mechanisms and movement dysfunction. One possible explanation may be that experienced pain is affecting overall activity and leading to more sedentary behavior and deconditioning, which may inform the compromised biomechanical function. Future work will examine activity patterns from actigraphy to understand how it may contribute to the relationship between pain experience and biomechanical function.

When bifurcating by sex, this association weakened for females (R^2^ = 0.05) but remained for males (R^2^ = 0.13), suggesting the lower pain sensitivity in males with LBP provides insight into the mechanistic relationship with biomechanical impairment. Two explanations for the observed link between higher PPT and reduced movement quality in males include (1) reduced interoceptive awareness, and (2) increased kinesiophobia. First, pain thresholds are an interoceptive process [38]. Research has previously highlighted that males generally have reduced interoceptive awareness compared to females [39], also supported by the observed lower PPT in males in this study. Reduced interoceptive awareness can impair postural control [40], supported by emerging therapeutic methods aimed at heightening awareness of the pain site [41]. Secondly, lower general pain sensitivity in males may result in heightened kinesiophobia and reduced physical function, as males may be less accustomed to discomfort. Many studies support this notion, underlining that although females report high pain, males with chronic musculoskeletal pain have intensified kinesiophobia [20–23], leading to lower activity levels, reduced pain acceptance, and maladaptive movement patterns [11, 22].

The study is limited by the small control sample size, limiting our ability to have a sex-matched control group. Additionally, the study included one activity and lacked validation for other functional tasks and actigraphy, which will be addressed in future studies. Selection bias is present, as severe LBP cases were excluded due to the requirement to complete motion tasks, reflected in the mild to moderate PASS-20 and PROMIS-PI scores (Table 1). The observational nature of the study introduces uncertainty regarding underlying causes of pain that could confound the analysis, including sex-based differences in the etiologies of LBP, such as variations in anatomical structure (e.g., pelvic alignment), hormonal influences (e.g., estrogen levels), or occupational exposures (e.g., repetitive physical tasks). Finally, the K-Score assumes that 'improved movement quality' aligns with the healthy control trajectory, but deviations from this path do not necessarily indicate maladaptation. Future work is needed to understand the implications of differences from healthy motion, as well as dissecting the K-Profiles and K-Scores to understand how patients deviate from healthy motion, allowing for a deeper exploration of biomechanical impairment.

## Conclusion

This study highlights sex-based differences influencing the relationship between pain sensitivity and biomechanical function in chronic LBP. Males exhibited greater deviations in movement from healthy controls despite higher pain thresholds, highlighting a difference in the relationship between biomechanics and pain sensitivity across sexes. The findings underscore the need to consider sex-specific mechanisms when investigating the intersection of pain and movement, which could contribute to effective patient-specific interventions for chronic LBP.

## Data Availability

The data that support the findings of this study are maintained and archived at The UCSF Core Center for Patient-centric Mechanistic Phenotyping in Chronic Low Back Pain (UCSF REACH). Data is available from the corresponding author, upon reasonable request, and with permission of UCSF REACH.
